# The effect of primary delivery of the anterior compared with the posterior shoulder on perineal trauma: a study protocol for a randomized controlled trial

**DOI:** 10.1186/1745-6215-15-291

**Published:** 2014-07-21

**Authors:** Hanne Willer, Anna JM Aabakke, Lone Krebs

**Affiliations:** 1 Department of Obstetrics and Gynaecology, University of Copenhagen, Holbæk Hospital, Holbæk, Denmark

**Keywords:** anal canal/injuries, delivery, obstetric/methods, delivery of the shoulders, labour stage, second, lacerations/prevention and control, obstetric labour complications/prevention and control, perineum/injuries

## Abstract

**Background:**

Approximately 85% of vaginal deliveries are accompanied by perineal trauma. The objective of this trial is to compare the incidence and degree of perineal trauma after primary delivery of the anterior compared with the posterior shoulder during vaginal birth. The hypothesis is that primary delivery of the posterior shoulder reduces the rate and degree of perineal trauma.

**Methods/design:**

This is a single-centre, randomized controlled trial, with computer-generated randomization in a 1:1 allocation ratio. Women planning their first vaginal delivery (*n* = 650) are randomized to primary delivery of either the anterior or posterior shoulder. The primary outcome is any perineal trauma. Additional outcomes are the perineal injury subtypes, postpartum bleeding, umbilical artery pH, Apgar score at 5 minutes and any neonatal birth trauma. Perineal trauma is assessed by a midwife or doctor blinded to the method of shoulder delivery. All midwives are trained in the two methods of shoulder delivery and in the grading of perineal tears. The trial is being undertaken at a Danish community hospital with 1,600 yearly deliveries. Data will be analyzed according to the intention-to-treat principle. Recruitment started in January 2013 and the trial is planned to proceed for 24 months.

**Discussion:**

Most delivery assistance techniques are based on tradition and heritage and lack objective evidence. This trial provides an example of how vaginal delivery techniques can be evaluated in a randomized controlled trial. The results of this trial will clarify the role that delivery of the shoulders has on perineal trauma and thereby provide knowledge to recommendations on birthing technique.

**Trial registration:**

ClinicalTrials.gov: NCT01937546.

## Background

Approximately 85% of vaginal deliveries are accompanied by trauma to the genital tract, with a higher risk at the first compared with subsequent vaginal births [1,2]. Among primipara with vaginal deliveries, 86% sustain a vaginal or perineal tear, and 77% require suturing of a lesion [1,3].

Birth trauma is associated with both short- and long-term morbidity, including pain, discomfort, dyspareunia and fecal incontinence, and perineal trauma may cause social problems and affect the psychological well-being of the mother [1,4]. The level of postpartum morbidity is related to the degree of trauma [5,6], and studies of preventive measures are therefore of interest.

Genital tract traumas are classified into subtypes according to the location and severity of the lesion. Most studies have evaluated the risk factors for third- and fourth-degree perineal tears that include the anal sphincter complex, so-called obstetric anal sphincter injuries (OASIS). Predisposing factors are increasing maternal age, heavier birthweight, longer duration of the second stage of labour, oxytocin augmentation, occiput posterior position and instrumental delivery [7-9]. The risk increases with the number of different risk factors [9]. Protective factors are previous vaginal delivery, epidural analgesia and multiparity [7-9]. Several perineal management techniques used during delivery have been studied, and a recent Cochrane review concluded that warm compresses and perineal massage seem to reduce the risk of OASIS [4]. The introduction of an interventional perineal protection programme also seems to reduce the incidence of OASIS [10-12].

Leading textbooks recommend primary delivery of the anterior shoulder by gentle traction if the shoulders are not delivered spontaneously [13,14]. However, if shoulder dystocia occurs, the recommended manoeuvres are primary delivery of the posterior arm or primary delivery of the posterior shoulder, with the woman positioned on her hands and knees (Gaskin’s manoeuvre) [15,16]. A computer-simulated trial of the manoeuvres used during shoulder dystocia found that primary delivery of the posterior arm caused an 80% reduction in the delivery force and a 70% reduction in stretch to the brachial plexus [17]. Primary delivery of the posterior shoulder could therefore be of advantage during uncomplicated deliveries, but, to the knowledge of the authors, various methods of shoulder delivery have never been studied previously.

The objective of this trial is to evaluate the incidence and degree of perineal trauma after primary delivery of the anterior shoulder compared with primary delivery of the posterior shoulder during vaginal birth in primiparous women in a randomized controlled trial. The hypothesis is that primary delivery of the posterior shoulder reduces the rate and degree of perineal trauma.

## Methods/design

### Setting

This is a single-centre, prospective, investigator-initiated, randomized controlled trial. The trial is being undertaken at the University of Copenhagen, Holbæk Hospital, which is a Danish community hospital with an obstetric unit with 1,600 yearly deliveries. Recruitment started 1 January 2013, and the trial is planned to proceed for 24 months.

### Participants

Eligible participants are primiparous women and women with one previous Cesarean section, who are planning a vaginal delivery of a fetus in cephalic presentation. Patients have to be able to provide informed oral and written consent. Exclusion criteria are multiparity with a previous vaginal delivery, multiple pregnancy, acute or planned Cesarean section, delivery before 35 weeks of gestation, and breech delivery.

### Interventions

**Figure 1:**
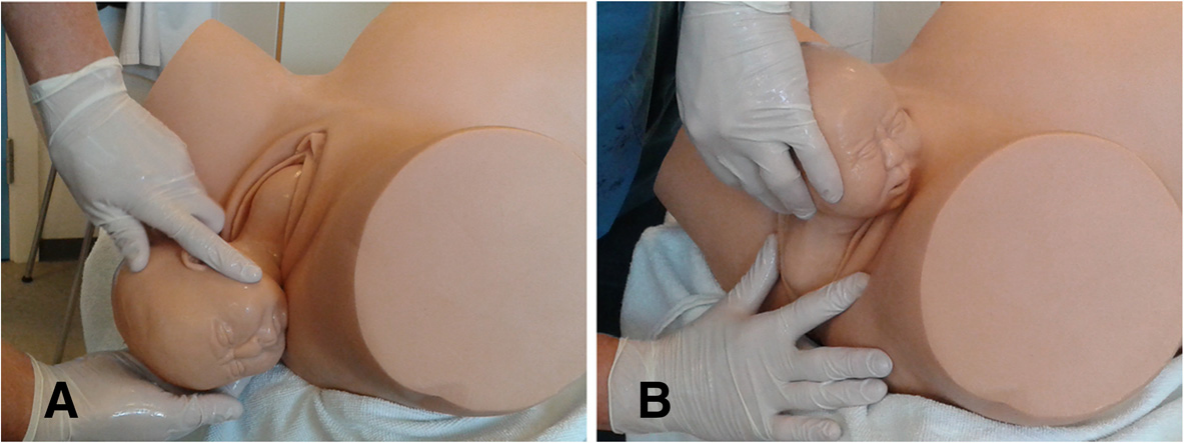
Primary delivery of (A) anterior shoulder and (B) posterior shoulder.

The trial interventions take place during vaginal birth after the delivery of the head. The head is supported in the spontaneous rotation that occurs after its delivery, and the shoulders are delivered according to randomization. The intervention is primary delivery of either the anterior or the posterior shoulder, as illustrated in Figure 1 and the additional movie files (see Additional file 1 and Additional file 2). It is performed by the midwife placing her hands around the head of the baby and applying gentle traction in the appropriate direction.

All midwives are trained in the two interventions by the primary investigator (HW) to secure uniform use of the techniques. Training sessions include an introductory video of the two methods and practical training on a birthing phantom (MODEL-med ‘Sophie and her Mum Full Birth Obstetric Trainer’, Carnegie, Australia). All midwives are also trained in evaluation and classification of perineal tears using an e-learning programme (GynZone ApS, Aarhus, Denmark). Primary training sessions (*n* = 14) took place from February to November 2013, and additional sessions are held every second month during the course of the trial to train new midwives and update previously trained midwives. Attendance at a training session accredits midwifes to deliver included patients.

The participants can deliver in the position they prefer, and if spontaneous delivery of the shoulders occurs, this is to be respected regardless of randomization. If during delivery the midwife judges that an alternative method of delivery is preferred with regard to the safety of the birthing mother, this overrules randomization. Episiotomy may be used in accordance to local guidelines, as judged necessary by the midwife. In case of vacuum-assisted delivery, which according to guidelines is performed by physicians, the midwife delivers the shoulders. Any deviations from the protocol are registered on the clinical registration form.

### Outcomes

The primary outcome is any perineal trauma. Secondary outcomes are the perineal injury subtypes, postpartum bleeding in millilitres evaluated 2 hours after birth, umbilical artery pH, Apgar scores at 5 minutes and neonatal birth trauma, including fractures of the clavicle and humerus, and brachial plexus injury.

Perineal traumas are classified as anterior or posterior, and posterior traumas are classified into subtypes. Anterior trauma comprises lacerations of the labia that do or do not require suturing. Posterior subtypes comprise lacerations of the vagina and/or perineum that do not require suturing; first-degree tears, where only the skin or mucosa is involved (1A: <1.5 cm; 1B: ≥1.5 cm); second-degree tears, where skin and perineal muscle is involved with the anal sphincter intact but not visible (2A) or visible (2B); third-degree tears involving the anal sphincter complex (3A, less than 50% of the external anal sphincter is torn; 3B, 50% or more of the external anal sphincter is torn; 3C, both the internal and external anal sphincters are torn); and fourth-degree tears involving the anal sphincter complex as well as anal epithelium [18,19].

### Assessment

After delivery of the placenta, a blinded midwife or doctor not otherwise involved in the delivery assesses the perineum and grades the perineal tears. Tears are sutured and officially classified and coded independently of the trial by the midwife responsible for the delivery or a doctor according to the hospital guidelines. Secondary outcome measures are registered by the midwife responsible for the delivery. Assessors of the primary outcome and the primary investigator are blinded to randomization.

Registered third- and fourth-degree tears are validated through manual assessment of patient records. We assume that higher-degree tears, in particular, may be diagnosed after trial assessment during repair. In order not to underestimate the level of higher-degree tears, data on all third- and fourth-degree tears in our study population registered in the hospital register during the trial period will be retrieved after the end of the trial. These data will be validated against patient records and any additional confirmed cases of third- or fourth-degree tears in our study population will be incorporated into the final dataset.

### Recruitment

**Figure 2:**
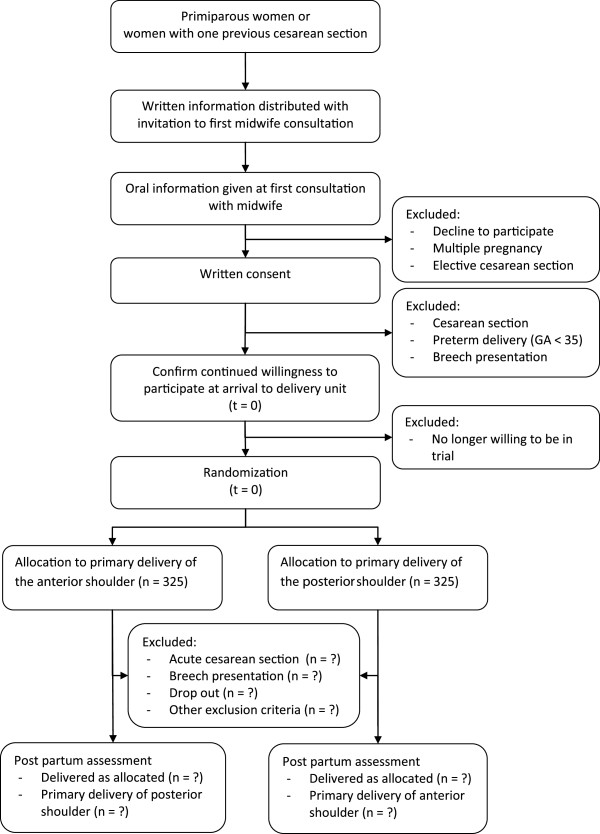
**Time schedule and flow of participants.** GA, gestational age (weeks); *t*, time.

Eligible women receive an invitation to participate and the written trial information by mail together with the invitation to the first midwife consultation. Oral information is given by the consulting midwife during the first midwife consultation (14 to 15 weeks of gestation). Written consent is given any time during the pregnancy and confirmed with the participant upon arrival at the delivery unit at the time of birth. Subsequently, consenting women are randomized. The flow of participants and the time schedule are illustrated in Figure 2.

### Randomization

Randomization is computer-generated, with a 1:1 allocation to primary delivery of the anterior or posterior shoulder by a third party not otherwise involved in the trial. The allocation is concealed in 650 identical, opaque, sequentially numbered sealed envelopes. The allocation list is stored electronically by a third party not otherwise involved in the trial. Recruited women are randomized after confirmation of consent upon arrival at the delivery ward and a random allocation envelope is drawn. The envelope is opened by the midwife when the patient enters the second stage of labour and is destroyed thereafter. The allocation is only shown to the midwife and the assistant, and if necessary the obstetrician. During delivery, the midwife and assistant observe what shoulder is delivered first, and this is registered on the clinical registration form.

### Data management

Data are recorded on clinical registration forms and entered into a SPSS database (SPSS Inc., Chicago, IL, USA) by the primary investigator. Data will be available only to the investigators and the data monitor. After completed follow-up, data will be cleaned and consecutively locked before the allocation is broken.

All clinical registration forms are identified by a coded ID number according to randomization, to maintain participant confidentiality. All study-related participant information is stored in a locked room at the study site. Lists linking participant ID numbers to personal identification numbers are stored separately. Trial data will be stored for 10 years. Data will subsequently be anonymized and personally identifiable information destroyed.

### Data monitoring

The steering committee consists of the authors of the protocol and Anne Fabricious, Head Midwife, Department of Obstetrics and Gynaecology, University of Copenhagen, Holbæk Hospital.

To ensure that it is not unethical to continue due to one intervention causing markedly more perineal trauma, interim analyses will be performed by a data monitor not otherwise involved in the trial after the first year and consecutively every 6 months until the end of the trial. In the interim analysis, the distribution of the two interventions will be estimated among cases with no perineal tears and cases with third- and fourth-degree tears. The steering committee will be notified if more than 60% of the cases with no tears or a higher-degree tear belong to a particular intervention group. The steering committee will then decide if the trial has to be stopped before completion.

### Statistics

An audit at a Danish University Hospital of all their primary vaginal deliveries from 2003 to 2011 (*n* = 15,587) found that 86% sustained a perineal tear [3]. The hypothesis of this trial is that primary delivery of the posterior shoulder can reduce the incidence of perineal tears, and that a reduction of 10% is clinically significant. It is assumed that the rate of perineal tears using the traditional method of delivering the anterior shoulder first is 85%. The sample size calculation for the primary outcome is based on a minimal relevant difference in the proportions of perineal tears between the two intervention groups of 10%, with 80% power and a significance level of 0.05. Thus, we estimated that 250 women would have to be included in each intervention arm. In Denmark, 18% of primiparous women had an acute Cesarean section in 2011 [20]. Because of this probability of acute Cesarean section and the likelihood of drop-outs, 650 women will be included in this trial.

At the University of Copenhagen, Holbæk Hospital, 840 children were delivered by primiparous women in 2011. With an inclusion rate of 80% and assessment starting in January 2013, it is estimated that randomization can be finalized before December 2014.

All included women delivering vaginally will be included in the final analysis. The analysis will primarily be based on an intention-to-treat principle. It will be supplemented with a per-protocol analysis (of the women who received their random allocation) and an as-treated analysis (according to how the shoulders were actually delivered). Categorical variables, including the primary outcome, will be analyzed with the chi-squared test or Fisher’s exact test as appropriate, and the odds ratios presented with 95% confidence intervals. Continuous variables will be assessed for normal distribution with the Kolmogorov-Smirnov test. When normally distributed, they will be analyzed with a *t* test, and when not, with a Mann-Whitney test.

### Ethical consideration

Gentle traction is recommended to deliver the shoulders during vaginal birth, and there is no available evidence of which shoulder should be delivered first. There is therefore no increased risk involved for the women included in the trial, while an increased focus on preventing perineal trauma through the trial may be advantageous for the participants. The results of the trial will improve future birthing care.

The trial complies with the current version of the Declaration of Helsinki on biomedical research. The trial is approved by the regional ethics committee for Region Zealand (registration number SJ 310) and is registered with ClinicalTrials.gov (registration number NCT01937546).

All included women will give written informed consent confirming that they have received adequate written and oral information. Trial participants do not receive any financial compensation.

We followed the SPIRIT (Standard Protocol Items: Recommendations for Interventional Trials) guidelines for writing clinical trial protocols [21]. The latest version of the trial protocol is available in Danish on the trial website [22]. Any amendments to the trial protocol have to be accepted by the regional ethics committee.

The results of the trial will be disseminated through presentation at international conferences and will be published in an international peer-reviewed medical journal regardless of magnitude and direction of effect. The authors of this protocol will co-author the final trial report. All authors of the final trial report will adhere to the criteria of the International Committee of Medical Journal Editors [23]. After publishing the final trial report, the final results of the trial will be published in Danish on the trial website, where the final trial report will also be made available.

## Discussion

The purpose of this randomized controlled trial is to compare two methods of delivering the shoulders during vaginal delivery, with perineal trauma as the primary outcome.

The literature on delivery techniques is limited, and previous studies have primarily focused on their effect on OASIS. Most studies have been non-randomized, and different shoulder delivery techniques have not been evaluated previously. Thus, the strengths of this study are the randomized design, the intervention studied, that is, the delivery of the shoulders at vaginal delivery and the outcome of any perineal trauma.

The validity of this trial could be affected by the fact that several midwives perform the interventions. However, numerous birth assistants are a reality at most centres, thereby increasing the external validity and generalizability of the trial. Additionally, it might be interpreted as a limitation that this is a single-centre trial, although it increases internal validity of the trial. The perineal tears are evaluated by several objective assessors (midwives or doctors), which might possibly affect the validity of the outcome assessment. We will try to overcome this issue by validating registered higher-degree tears (third- and fourth-degree) by examining patient records. Additionally, we are planning to retrieve data on all third- and fourth-degree tears in our study population during the study period from the hospital registers and validate these data against patient records. We assume that higher-degree tears, in particular, may be diagnosed after trial assessment. These tears will be registered centrally because registration commonly takes place after lesion repair. Thus, all higher-degree tears included in our final analysis will be validated.

Most delivery assistance techniques used today are based on tradition and heritage and are not evidence-based. This trial provides an example of how vaginal delivery techniques may be evaluated in a randomized controlled trial.

The results of this trial will clarify the role that delivery of the shoulders has on perineal trauma and thereby provide knowledge to recommendations on birthing technique.

## Trial status

Recruitment commenced in January 2013, and the first included patient delivered in June 2013. The trial is currently recruiting.

## Abbreviations

OASIS: obstetric anal sphincter injuries; SPIRIT: Standard Protocol Items: Recommendations for Interventional Trials.

## Competing interest

The authors declare that they have no competing interests.

## Authors’ contributions

HW conceived the study and participated in its design, coordination and data acquisition. AJMA participated in the design of the study and drafted the manuscript. LK participated in the design and coordination of the study. All authors have revised the manuscript critically, and read and approved the final manuscript.

## Authors’ information

HW is a registered midwife and primary investigator of the trial. AJMA is a physician and LK is a physician and DMSc.

## Supplementary Material

Additional file 1 Primary delivery of the anterior shoulder. Click here for file

Additional file 2 Primary delivery of the posterior shoulder. Click here for file
